# Veterinary Department, Columbian University

**Published:** 1897-05

**Authors:** 


### VETERINARY DEPARTMENT, COLUMBIAN UNIVERSITY.

The commencement exercises of this school were held April 10th, in the lecture hall of the University. On the stage were seated President Whitman and the Faculty of the school, with the ten graduates. After an overture by the orchestra, Rev. John O. Knott offered prayer. Prof. D. E. Salmon addressed the students, discussing the progress in medical science and its application to animals. The valedictory was delivered by Mr. Elbert C. Switzer, after which President Whitman presented diplomas to the following graduates: Reid R. Ashworth, of Rhode Island; William H. Boyln, of Virginia; Basil A. Brown, of England; William P. Ellenberger, of Illinois; Joseph Neilson Megary, of Maryland; Floyd G. Scannel, of New York; John Shaw, of Delaware; Elbert C. Switzer, of Massachusetts; Robert H. Twitty, of North Carolina; George Ransom White, of Tennessee. Dr. Whitman, in his closing remarks to the graduates, stated that the University would follow their careers with interest. They would always find a cordial welcome there.

The following gentlemen obtained a high general average in their examinations and were given honorable mention: Reid R. Ashworth, Elbert C. Switzer, and George R. White.

